# Can convertible metal-backed glenoid components replace cemented polyethylene glenoid components in anatomical total shoulder arthroplasty?

**DOI:** 10.1186/s12893-023-02092-6

**Published:** 2023-07-05

**Authors:** Myung-Sun Kim, Yeong-Seub Ahn, Sun-Ho Lee, Hyeon Jang Jeong, Young Kyu Kim, Joo Han Oh

**Affiliations:** 1 Department of Orthopaedic Surgery, Chonnam National University College of Medicine, Chonnam National University Hospital, Gwangju, Republic of Korea; 2 Department of Orthopaedic Surgery, Seoul National University College of Medicine, Seoul National University Bundang Hospital, Seongnam, Republic of Korea; 3 Department of Orthopaedic Surgery, Good Morning General Hospital, Pyeongtaek, Republic of Korea; 4 Department of Orthopaedic Surgery, Daejin Medical Center Bundang Jesaeng General Hospital, Seongnam, Republic of Korea

**Keywords:** Cemented polyethylene component, Convertible metal-backed component, Anatomical total shoulder arthroplasty, Revision arthroplasty

## Abstract

**Background:**

Anatomical total shoulder arthroplasty (aTSA) has been used to manage degenerative diseases such as primary osteoarthritis. An increase in the use of this procedure has led to several developments in humeral and glenoid components to improve patient outcomes. This study aimed to compare clinical and radiological outcomes of the newly-introduced convertible metal-backed glenoid components with cemented polyethylene glenoid components in aTSA, and to determine whether the new component would be comparable to a conventional one for reducing the burden of future revision or conversion surgeries.

**Methods:**

Medical records of fifty patients who underwent aTSA with at least two years of follow-up were retrospectively reviewed. Eighteen patients received convertible metal-backed glenoid components with vitamin E1-coated liner (MB group), while thirty-two patients received conventional cemented polyethylene glenoid components (PE group). Pre- and postoperative clinical and radiological outcomes (acromion-greater tuberosity angle [AGA] and humeral lateral offset [LO]) at final follow-up were assessed. Radiolucent lines (RLLs) and loosening around the humeral and glenoid components were also evaluated.

**Results:**

Clinical outcomes improved after surgery in both groups (all *p* < 0.001). The arc of rotation measured by AGA improved postoperatively in both groups (all *p* < 0.001), and AGA and LO were not different according to the type of glenoid components (all *p* > 0.05). Overall complication rates including RLLs of PE and MB groups were 43.8% (14/32) and 16.7% (3/18), respectively (*p* = 0.031). Although the PE group had more RLLs than did the MB group (*p* < 0.05), related symptoms and/or glenoid implant loosening were not observed in both groups. Subscapularis failure occurred in two patients in the PE group and in one in the MB group.

**Conclusion:**

The convertible metal-backed glenoid implant with vitamin E1-coated liner may be a good alternative for considering the potential for an easier conversion to reverse total shoulder arthroplasty.

**Supplementary Information:**

The online version contains supplementary material available at 10.1186/s12893-023-02092-6.

## Introduction

Anatomical total shoulder arthroplasty (aTSA) has been an effective tool to manage degenerative diseases including primary osteoarthritis [1]. As the annual number of aTSA is increasing dramatically, several developments in the humeral and glenoid components have been made with various outcomes [2, 3]. However, despite advances in implant design with various surgical techniques, cemented polyethylene (PE) glenoid component has been the gold standard of treatment for glenoid replacement in aTSA.

Despite the successful use of cemented PE in aTSA, glenoid component failure is still one of the most frequently occurred complications after aTSA. To increase the stability of the glenoid component, cementless metal-backed (MB) glenoid devices have been tried. However, the 1st generation MB glenoid components [4] in aTSA have been greatly criticized because of increased periprosthetic loosening, dissociation, and early PE wear, which already progressed significantly within three years [5], and led to a revision rate three times higher than that with conventional cemented PE components [5–10]. Furthermore, increased glenoid component thickness induced by the metal tray [11, 12] could be a risk factor for rotator cuff insufficiency [13, 14].

Although clinical outcomes of the cemented PE glenoid components were shown to be acceptable [5, 15], 20% of patients require revisions before the 15th year of follow-up because of glenoid component loosening and/or rotator cuff failure [15, 16]. Furthermore, conversion to a reverse total shoulder arthroplasty (rTSA) from an aTSA owing to rotator cuff failure has increased according to the aging process in long-term follow-up. However, the extraction of the well-fixed cemented PE glenoid component might provoke severe glenoid bone defects, which force the bone graft to provide primary stability for the fixation of the rTSA glenoid component. Therefore, there is an increasing need for conversion to rTSA without changing the glenoid component.

Recently, 2nd generation trabecular MB components with convertible and modular parts have been introduced. This newly developed convertible MB system of aTSA is designed to enhance the fixation to the glenoid bone contact area. Theoretically, the porous-coated baseplate induces osseous integration to enhance biologic fixation in the implant-bone interface, and the compressive force of the baseplate against the glenoid could be obtained with peripheral screws. Furthermore, new vitamin E1-coated liner could increase endurance against wear, compared to conventional PE. In a conversion setting, the PE can be easily removed and changed to the glenosphere without baseplate removal. Therefore, bone loss during extraction of the glenoid component might be decreased compared with the conventional cemented PE component.

However, to the best of our knowledge, there are no previous studies that have directly compared a cemented PE glenoid to a newly developed convertible MB system of aTSA. Therefore, we aimed to compare the clinical and radiological outcomes of a new convertible MB glenoid component with a conventional cemented PE glenoid system among patients who followed up for a minimum of two years. Authors hypothesized that the newly invented 2nd generation convertible MB glenoid component would present favorable clinical and radiological outcomes compared to that of the cemented PE glenoid component.

## Materials and methods

### Ethics statements

This study was approved by the local institutional review board (reference number: Chonnam National University Hospital ; 2020 − 374 / Seoul National University Bundang Hospital ; B-2107/698 − 105).

### Study design and population

We retrospectively reviewed the medical records of patients who underwent aTSA to treat arthritic changes in the glenohumeral joint without a rotator cuff tear, including osteoarthritis, rheumatoid arthritis, and/or avascular necrosis, in two tertiary hospitals between 2012 and 2019. To minimize the heterogeneity originating from the different designs of the aTSA, we included patients with a single type of cemented PE or cementless convertible MB glenoid component using the Comprehensive System^®^ (Zimmer-Biomet, Warsaw, IN, USA).

To decrease the effect of confounders, patients who suffered rotator cuff insufficiency preoperatively (n = 12), and/or had previous surgical history including rotator cuff repair on the ipsilateral shoulder (n = 8), were excluded. Of the 70 patients whose data were screened, 50 were finally enrolled in the analyses.

Patients were classified into two groups according to the type of glenoid implant. A conventional cemented PE glenoid component was used in 32 patients (PE group), while a cementless MB glenoid component with vitamin E1-coated liner was used in the remaining 18 patients (MB group).

Clinical and radiological outcomes were evaluated at 4 weeks, 12 weeks, 6 months, 1 year after surgery, and every annually thereafter beginning from postoperative 1 year. The mean age at surgery was not significantly different between the two groups (*p* = 0.053), and the mean follow-up period for all patients was 43 ± 20.1 months (Table 1).

**Table 1:** Demographic data for the cemented polyethylene (PE) and convertible metal-backed (MB) groups

	Cemented PE group (n = 32)	Convertible MB group (n = 18)	*p*-value
Age at surgery, years	66.9 ± 11.2	60.6 ± 10.4	0.053
Sex, M:F	8:24	8:10	0.157
Site, right/left	18:14	10:8	0.962
Follow-up, months	49.3 ± 21.8	32.6 ± 10.8	0.002^*^
ASA physical status score	1.9 ± 0.7	1.9 ± 0.2	0.895

Data are presented as mean ± standard deviation

* Statistically significant

M: male, F: female, ASA: American Society of Anesthesiologists

### Functional evaluation

To compare the functional outcomes between the PE and MB groups, we evaluated the active range of motion (ROM), including forward flexion, external rotation, and internal rotation; the visual analog scale for pain (pVAS); American Shoulder and Elbow Surgeons (ASES) score; Simple Shoulder Test (SST); and Disabilities of the Arm, Shoulder, and Hand (DASH) score preoperatively, and at the final follow-up visit. The forward flexion and external rotation in a neutral arm position were estimated using a goniometer, and the internal rotation (IR) at the back was determined by numbering the spinous process where the tip of the patient’s ipsilateral thumb could reach [17].

### Radiological evaluation

The height (HH) and diameter (HD) of the humeral head implant, the acromion-greater tuberosity angle (AGA), and the lateral offset of the humerus (LO) were measured using the Grashey view of plain radiographs preoperatively and at the final follow-up. These parameters were compared with those of the contralateral shoulder without pathological changes pre- and postoperatively, and the differences according to the type of glenoid implant were also evaluated.

**Fig. 1:**
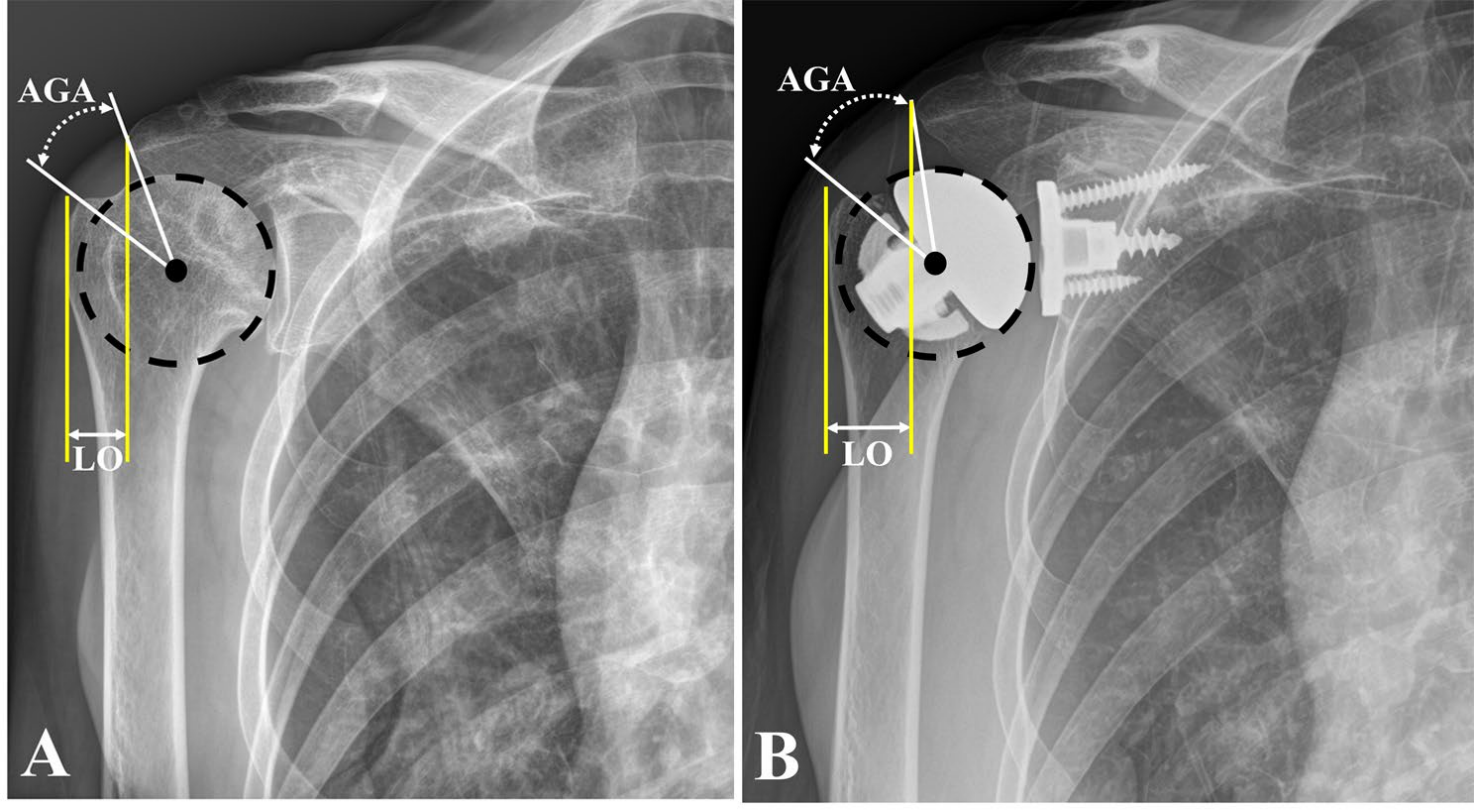
The acromion-greater tuberosity angle (AGA) is measured at the intersection angle created by two lines from the center of the humeral head or humeral head implant to the acromion and greater tuberosity. The degree of lateralization is measured by the lateral offset (LO) of the humerus. The LO of the humerus is measured based on the distance between the vertical line from the lateral border of the acromion to the most lateral area of the greater tuberosity marked by LO. In a radiologic comparison of preoperative **(a)** and postoperative **(b)** status, both AGA and LO are increased

The AGA was radiologically assessed to evaluate the difference in the center of the rotation arc (Fig. 1). As the humeral head moves superiorly and medially owing to arthritic changes, the center of rotation moves medially, and the rotation arc angle measured by the AGA decreases. A comparison with the contralateral shoulder without pathology, and a comparison of the condition before and after surgery, was performed. The AGA was measured at the intersection angle created by two lines from the center of the humeral head or humeral head implant to the acromion and greater tuberosity (GT). The degree of lateralization, which is thought to be the cause of cuff failure, was evaluated by the LO that was measured based on the distances presented by the vertical line from the lateral border of the acromion to most of the lateral GT area (Fig. 1).

**Fig. 2:**
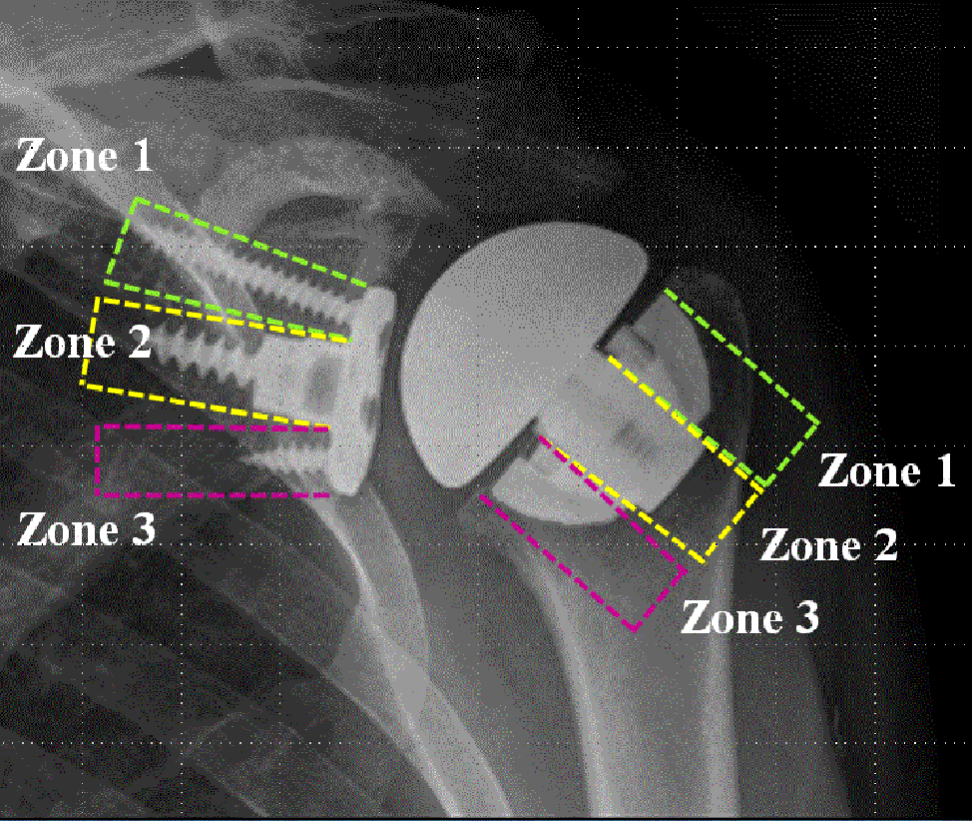
Radiolucent lines (RLL) for evaluation of loosening of the implant. To assess the RLL, implant-bone interface of glenoid and humerus are divided into three different zones in both anteroposterior view (AP) and axillary view of the plain radiograph. In glenoid, zone 1, zone 2 and zone 3 are defined as implant-bone interface around the superior (AP) or anterior (axillary view) peripheral screw, central screw, and inferior (AP) or posterior (axillary) peripheral screw, respectively. Similar to glenoid implant-bone interface, humeral implant-bone interface is evenly divided based on the peg of humeral tray

To evaluate the loosening of the implant, radiolucent lines (RLLs) were assessed using serial plain radiographs including anteroposterior and axillary views during the study period by dividing the implant-bone interface into three different zones (Fig. 2) [18].

### Surgical procedure and the rehabilitation protocol

All surgeries were performed using a deltopectoral approach. A subscapularis tenotomy was performed in all patients, the subscapularis tendon was reattached using a transosseous repair technique. The long head of the biceps underwent tenodesis with a suture to a transverse humeral ligament. To decrease the heterogeneity of the various designs of the implants, each of the PE and MB groups used a single type of implant.

All patients underwent the same rehabilitation program. During 4 weeks of protective immobilization in an abduction brace, the patients started progressive passive-assisted ROM exercises followed by active-assisted ROM exercises for 12 weeks postoperatively. After the strengthening exercise period (until 6 months postoperatively), patients began sporting activities.

### Statistical analysis

Statistical analyses were performed using SPSS (version 21.0; IBM Corp., Armonk, NY, USA). The Shapiro–Wilk normality test was conducted for continuous variables. The paired t-test or Wilcoxon signed-rank test was used to compare pre- and post-operative clinical and radiological data. Analyses between the two groups were performed using an independent t-test or the Wilcoxon rank-sum test. The chi-squared test or Fisher’s exact test was conducted for categorical variables. A *p*-value of < 0.05 was considered statistically significant.

## Results

### Clinical outcomes

The pVAS, ROMs, and functional outcomes including ASES score, SST, and DASH score were not significantly different between the two groups (all *p* > 0.05, Table 2), and these were significantly improved after surgery in both groups (all *p* < 0.001, Table 2). Functional outcomes at the final follow-up were also not significantly different according to the type of glenoid implant (all *p* > 0.05, Table 2).

**Table 2:** Comparison of preoperative and final follow-up functional outcomes between the cemented polyethylene (PE) and convertible metal-backed (MB) groups

	Cemented PE group (n = 32)	Convertible MB group (n = 18)	*p*-value
Follow-up, months	49.3 ± 21.8	32.6 ± 10.8	0.002^*^
Pain, VAS			
Preoperative	7.0 ± 1.9	6.9 ± 1.3	0.805
Final follow-up	1.5 ± 2.2	0.5 ± 1.1	0.171
*p*-value	< 0.001^*^	< 0.001^*^	
Forward flexion, °			
Preoperative	110.9 ± 32.7	121.2 ± 33.7	0.199
Final follow-up	153.3 ± 15.9	158.1 ± 18.9	0.058
*p*-value	< 0.001^*^	< 0.001^*^	
External rotation, °			
Preoperative	29.6 ± 14.1	31.5 ± 11.7	0.385
Final follow-up	64.6 ± 16.9	67.6 ± 11.5	0.307
*p*-value	< 0.001^*^	< 0.001^*^	
Internal rotation at back, VL			
Preoperative	14.1 ± 2.9	13.3 ± 3.1	0.344
Final follow-up	9.6 ± 2.4	9.2 ± 2.0	0.862
*p*-value	< 0.001^*^	< 0.001^*^	
ASES score			
Preoperative	37.7 ± 18.6	42.5 ± 8.7	0.078
Final follow-up	87.2 ± 15.0	91.8 ± 12.3	0.246
*p*-value	< 0.001^*^	< 0.001^*^	
SST			
Preoperative	2.8 ± 2.3	3.1 ± 1.6	0.334
Final follow-up	8.6 ± 3.9	9.6 ± 2.6	0.546
*p*-value	< 0.001^*^	< 0.001^*^	
DASH score			
Preoperative	55.7 ± 22.9	46.2 ± 19.1	0.213
Final follow-up	10.8 ± 15.1	8.7 ± 8.9	0.999
*p*-value	< 0.001^*^	< 0.001^*^	

Data are presented as mean ± standard deviation

* Statistically significant, ° Degrees

VAS: visual analog scale, VL: vertebral level, ASES: American Shoulder and Elbow Surgeons Score, SST: simple shoulder test, DASH: Disabilities of the Arm, Shoulder, and Hand

To compensate for the difference in the follow-up period between the two groups, we conducted an additional study comparing the data of the two groups at about postoperative two years. In the results of this comparative study, it was confirmed that the functional outcomes at two years postoperative period showed a simillar pattern to the functional outcomes at the final follow-up period. The functional outcomes were not significantly different between the two groups (all *p* > 0.05, Table 3), and these were significantly improved after surgery in both groups (all *p* < 0.001, Table 3). Functional outcomes at the two year follow-up period were also not significantly different according to the type of glenoid implant (all *p* > 0.05, Table 3).

**Table 3:** Comparison of preoperative and two years postoperative period of functional outcomes between the cemented polyethylene (PE) and convertible metal-backed (MB) groups

	Cemented PE group (n = 32)	Convertible MB group (n = 18)	*p*-value
Follow-up, months	26.8 ± 3.0	26.7 ± 2.5	0.902
Pain, VAS			
Preoperative	7.0 ± 1.9	6.9 ± 1.3	0.805
2-year follow-up	1.5 ± 2.2	0.5 ± 1.1	0.119
*p*-value	< 0.001^*^	< 0.001^*^	
Forward flexion, °			
Preoperative	110.9 ± 32.7	121.2 ± 33.7	0.199
2-year follow-up	144.8 ± 26.3	156.8 ± 21.9	0.056
*p*-value	< 0.001^*^	< 0.001^*^	
External rotation, °			
Preoperative	29.6 ± 14.1	31.5 ± 11.7	0.385
2-year follow-up	66.5 ± 17.1	69.2 ± 12.5	0.552
*p*-value	< 0.001^*^	< 0.001^*^	
Internal rotation at back, VL			
Preoperative	14.1 ± 2.9	13.3 ± 3.1	0.344
2-year follow-up	9.6 ± 2.4	9.4 ± 2.1	0.760
*p*-value	< 0.001^*^	< 0.001^*^	
ASES score			
Preoperative	37.7 ± 18.6	42.5 ± 8.7	0.078
2-year follow-up	87.2 ± 15.0	91.8 ± 12.3	0.246
*p*-value	< 0.001^*^	< 0.001^*^	
SST			
Preoperative	2.8 ± 2.3	3.1 ± 1.6	0.334
2-year follow-up	8.1 ± 3.9	9.9 ± 2.7	0.245
*p*-value	< 0.001^*^	< 0.001^*^	
DASH score			
Preoperative	55.7 ± 22.9	46.2 ± 19.1	0.213
2-year follow-up	11.0 ± 15.2	8.7 ± 9.0	0.959
*p*-value	< 0.001^*^	< 0.001^*^	

Data are presented as mean ± standard deviation

* Statistically significant, ° Degrees

VAS: visual analog scale, VL: vertebral level, ASES: American Shoulder and Elbow Surgeons Score, SST: simple shoulder test, DASH: Disabilities of the Arm, Shoulder, and Hand

### Radiological outcomes

The HH and HD of the contralateral side were not significantly different in both groups (all *p* > 0.05, Table 4). In the PE group, HH of the affected side and contralateral shoulder without pathological change was not significantly different (*p* = 0.639, Table 4). However, the HD of the PE group, and the HH and HD of the MB group were significantly smaller than those of the contralateral shoulder (all *p* < 0.05, Table 4). Additionally, HH and HD of the affected side were significantly smaller in the MB group than in the PE group (all *p* < 0.05, Table 4).

**Table 4:** Comparison of radiological parameters between the cemented polyethylene (PE) and convertible metal-backed (MB) groups

	Cemented PE group	Convertible MB group	*p*-value^*^
Humeral head diameter (HD), mm			
Operation side	44.1 ± 3.4	41.5 ± 3.3	0.011^*^
Contralateral side	48.4 ± 3.4	48.2 ± 3.9	0.765
*p*-value	< 0.001^*^	0.002^*^	
Humeral head height (HH), mm			
Operation side	21.0 ± 2.1	19.7 ± 1.8	0.041^*^
Contralateral side	21.6 ± 1.7	21.7 ± 1.5	0.736
*p*-value	0.639	0.019^*^	
AGA, °			
Preoperative variables	28. 5 ± 10.5	28. 3 ± 8.3	0.685
Postoperative variables	44.5 ± 10.3	48.6 ± 8.9	0.149
*p*-value	< 0.001^*^	< 0.001^*^	
Humeral LO, mm			
Preoperative variables	9.7 ± 6.4	9.2 ± 6.1	0.779
Postoperative variables	17.3 ± 5.1	17.1 ± 6.1	0.982
*p*-value	< 0.001^*^	< 0.001^*^	

Data are presented as mean ± standard deviation

* Statistically significant, ° Degrees

AGA, acromion-greater tuberosity angle; LO, lateral offset

As the center of rotation was medialized because of arthritic changes, preoperative AGA was significantly lower in both PE and MB groups, compared with the contralateral shoulder (all *p* < 0.05, Table 5). However, AGA in both groups was increased after surgery (all *p* < 0.001, Table 5), and it was also larger than that of the contralateral shoulder (all *p* < 0.05, Table 5).

**Table 5:** Radiological comparison of the contralateral side of cemented polyethylene (PE) and convertible metal-backed (MB) groups

	Operation side	Contralateral side	*p*-value^*^
Cemented PE group AGA, °			
Preoperative variables	28. 5 ± 10.5	35.2. ± 7.1	< 0.001^*^
Postoperative variables	44.5 ± 10.3	35.2. ± 7.1	< 0.001^*^
*p*-value	< 0.001^*^		
Humeral LO, mm			
Preoperative variables	9.7 ± 6.4	12.8 ± 4.4	0.035^*^
Postoperative variables	17.3 ± 5.1	12.8 ± 4.4	0.003^*^
*p*-value	< 0.001^*^		
Convertible MB group AGA, °			
Preoperative variables	28.3 ± 8.3	38.2. ± 5.6	0.002^*^
Postoperative variables	48.6 ± 8.9	38.2. ± 5.6	0.002^*^
*p*-value	< 0.001^*^		
Humeral LO, mm			
Preoperative variables	9.2 ± 6.1	13.1 ± 3.9	0.008^*^
Postoperative variables	17.1 ± 6.1	13.1 ± 3.9	0.019^*^
*p*-value	< 0.001^*^		

Data are presented as mean ± standard deviation

* Statistically significant, ° Degrees

AGA, acromion-greater tuberosity angle; LO, lateral offset

The LO in both groups was also increased after surgery as the center of rotation became more lateralized (all *p* < 0.001, Table 5). In comparison with the contralateral shoulder, the preoperative LO was significantly lower on the affected side (all *p* < 0.05, Table 5). Similar to the AGA, the postoperative LO of the affected side was also larger than that of the contralateral shoulder (all *p* < 0.05, Table 5). The LO of the cemented PE group increased by approximately 7.6 mm (*p* < 0.001, Table 5), while that of the convertible MB group, which was thought to have a thicker glenoid component than the cemented PE group owing to the additional metal-backed component including the PE liner, increased by approximately 7.7 mm (*p* < 0.001, Table 4). The AGAs and LOs were not significantly different in both groups, pre- and postoperatively (all *p* > 0.05, Table 4).

### Complications and revision

Overall complication rates including RLLs of PE and MB groups were 43.8% (14/32) and 16.7% (3/18), respectively (*p* = 0.031). There were no RLLs or complications associated with the humeral component in either the cemented PE or the convertible MB groups. However, RLLs adjacent to the glenoid component were more frequently observed in the PE group (37.5%, 12/32) than in the MB group (11.1%, 2/18; *p* = 0.033). All RLLs were less than 2 mm, and it was not enlarged according to time progression. Radiolucent line-related symptoms and/or revision surgery associated with loosening were not observed in both group. Subscapularis failure occurred in two patients in the PE group and one in the MB group. Patients of the PE group did not complain of discomfort; therefore, subscapularis failure was managed conservatively. For patients in the convertible MB group, conversion to rTSA preserving the glenoid component and humeral stem was performed (Supplement). There were no other complications related to infection, bleeding, nerve damage, or periprosthetic fracture associated with the two implants included in this study.

## Discussion

In the current study, the clinical outcomes and radiological parameters of newly-introduced convertible MB glenoid components of aTSA were comparable to those of the conventional PE glenoid components. Furthermore, RLLs that might develop into loosening were less frequently observed in this 2nd generation convertible MB system than in PE glenoid components, although RLL-related symptoms were not observed in either group.

Despite the controversy regarding long-term outcomes, aTSA is still a reasonable treatment option for arthritis in the glenohumeral joint [5, 19]. Several previous studies have reported reliable clinical outcomes of aTSA with the conventional cemented PE-type glenoid implant [6, 8, 20]. However, one of the serious drawbacks of aTSA with a PE glenoid component is that patients are more likely to require revision surgery owing to glenoid loosening and/or rotator cuff insufficiency.

With the innovation of material engineering and the design of MB glenoid implants, a low prevalence rate of RLLs occurring at the implant-bone interface has been reported in 2nd generation MB glenoid components [5, 7, 11, 18, 20–23]. Similar to the previous studies, this study also confirmed a lower prevalence of RLLs in the MB group than in the PE group.

**Fig. 3:**
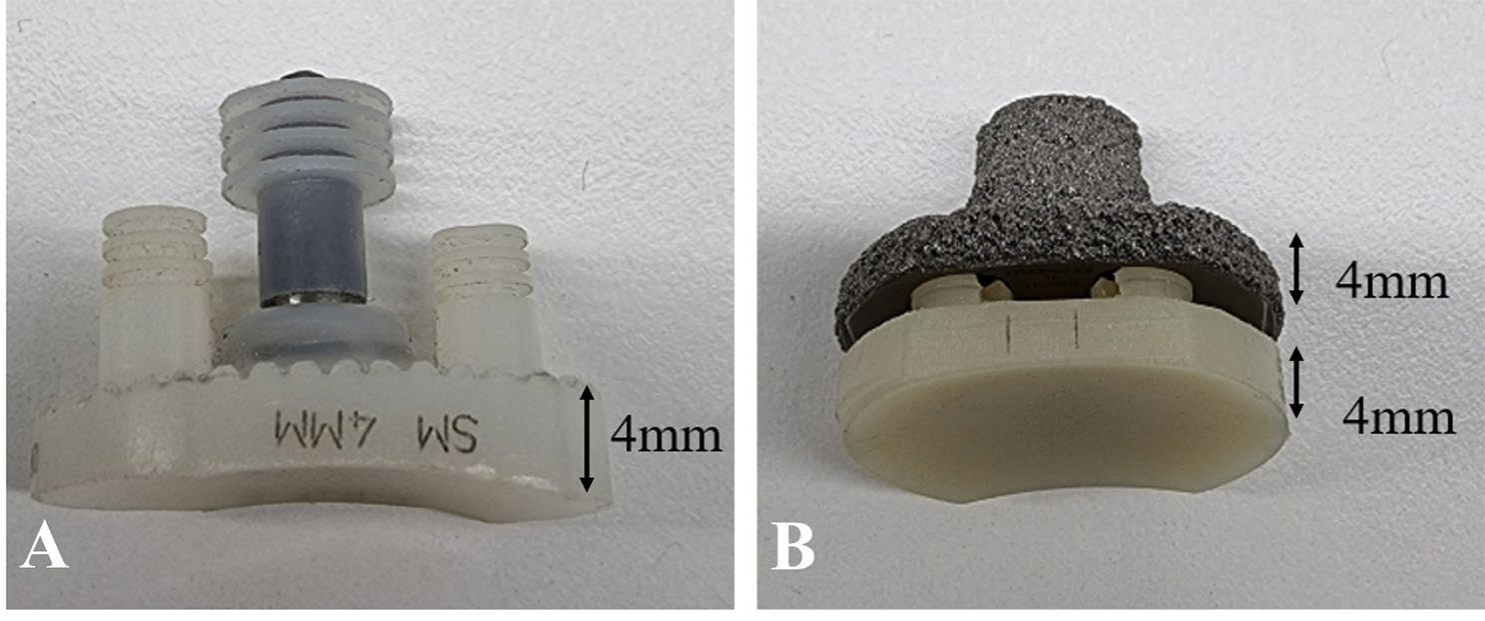
**(a)** The thickness of the polyethylene system without the metal-backed baseplate is approximately 4 mm. **(b)** Mechanism of the convertible metal-backed system, in which the metal baseplate is firmly fixed to the glenoid before polyethylene is inserted thereon, inevitably requires a thicker glenoid component than do the conventional polyethylene glenoid components

Despite the low prevalence of RLLs, other types of failure associated with the MB system have been reported [5, 6, 21, 22]. Metal-backed glenoid components were composed of a baseplate and PE liner. Therefore, the total thickness of the MB glenoid component was usually thicker than that of the PE glenoid component (Fig. 3). The thicker metal-backed glenoid components increase the risk of PE liner wear by stress concentration and differences in elasticity among the PE, metal, and bone interface [24–28], and cause soft tissue failure due to over lateralization of rotator cuff tendon [5, 29].

To prevent wear of the PE liner, a vitamin E1-coated PE liner was introduced. The wear rate of vitamin E1-coated PE liner was significantly decreased compared to conventional PE liner, even in weight-bearing joints such as the hip and/or knee [17, 21, 30]. Considering that the shoulder is a non-weight-bearing joint, we assumed that the wear resistance of vitamin E1-coated PE will be much more effective than that previously reported in other weight-bearing joints [17, 30–32]. In the current study, the follow-up duration of a new convertible MB glenoid component was relatively shorter than that of the PE glenoid component owing to the development of time sequence. However, considering the systematic review in which MB glenoid loosening was reported at the highest rate in the follow-up study within 36 months [33], it is thought that the complication rate can be compared even with a relatively short-term study. Therefore, we assumed that the data of this study presented meaningful results despite the short average follow-up period.

Another concern in MB glenoid implants is rotator cuff insufficiency by increased soft tissue tension because of the over-lateralization originating from the thicker glenoid components [5, 29]. To prevent rotator cuff insufficiency due to increased load on the joint, we tried to minimize the stress applied to the cuff and joint during implantation before repairing the subscapularis tendon. As a result, in this study, the joint load stress might be reduced by using a humeral head implant that was statistically smaller than that of the original humeral head. Consequently, the degree of lateralization was not significantly different in both conventional PE and convertible MB groups. In the present study, one case of failure of the subscapularis tendon in the MB group was identified and managed by conversion to rTSA. rTSA conversion was performed relatively easily by changing to a glenosphere after PE extraction while preserving the glenoid component and humeral stem during surgery because of the capability of the convertible platform system (Supplement). Considering the convenience of revision surgery, this new convertible MB system is thought to be a potential solution to reduce the burden of revision surgery.

This study has several limitations. First, we could not exclude the possibility of a selection bias owing to the retrospective nature of this study. Second, the heterogeneity originating from the two orthopedic surgeons in two institutions could affect the outcomes of this study. However, the influence of different surgeons on the results was probably mild since a senior surgeon with a sufficient understanding of this new convertible system at each institution performed the operation. When the surgical results of the patients performed by two surgeons were compared clinically and radiologically, it was confirmed that there was no significant difference (all *p* > 0.05). Third, due to the time sequence that a new convertible MB system was invented later, there is a limitation that the follow-up period was shorter and the number of patients included was smaller than cemented PE group. Though we conducted an additional study comparing the data of the two groups at about postoperative 2 years to compensate for the difference in the follow-up period between the two groups, additional further long-term clinical and radiological studies are mandatory to clarify the hypothesis.

## Conclusions

In this study, we observed favorable clinical and radiological outcomes of a new convertible MB glenoid component with vitamin E1-coated PE for aTSA. In addition to these results, considering the ease of revision surgery for the new convertible MB system and the fact that cemented PE is not suitable for revision surgery, it is thought that the new convertible MB system can be a new alternative. However, further long-term clinical and radiological observations are required.

## Electronic supplementary material

Below is the link to the electronic supplementary material.

Additional File 1: In the case of converting anatomical total shoulder arthroplasty (aTSA) to reverse total shoulder arthroplasty using a convertible metal-backed (MB) glenoid component system due to aTSA failure, the humeral head and polyethylene (PE) were easily removed without causing any further damage to the glenoid bone. Subsequently, the glenosphere was easily inserted onto the retained MB base plate, and the humeral tray with PE was replaced without any difficulties.

## Data Availability

The datasets used and/or analyzed during the current study are available from the corresponding author on reasonable request.

## Abbreviations

AGA: acromion-greater tuberosity angle
ASES: American Shoulder and Elbow Surgeons; anatomical total shoulder arthroplasty
GT: greater tuberosity
HD: diamter of the humeral head implant
HH: height of the humeral head implant
IR: internal rotation
LO: lateral offset
MB: metal-backed
PE: polyethylene
pVAS: visual analog scale for pain
SGML: Standard generalized markup language
RLLs: radiolucent lines
ROM: range of motion
rTSA: reverse total shoulder arthroplasty
SST: Simple Shoulder Test
