# Supplementary material for: Can convertible metal-backed glenoid components replace cemented polyethylene glenoid components in anatomical total shoulder arthroplasty?
Source: BMC Surg. 2023 Jul 5;23:193. doi: 10.1186/s12893-023-02092-6 (PMC10324271; doi:10.1186/s12893-023-02092-6)

**Figure 1**

**
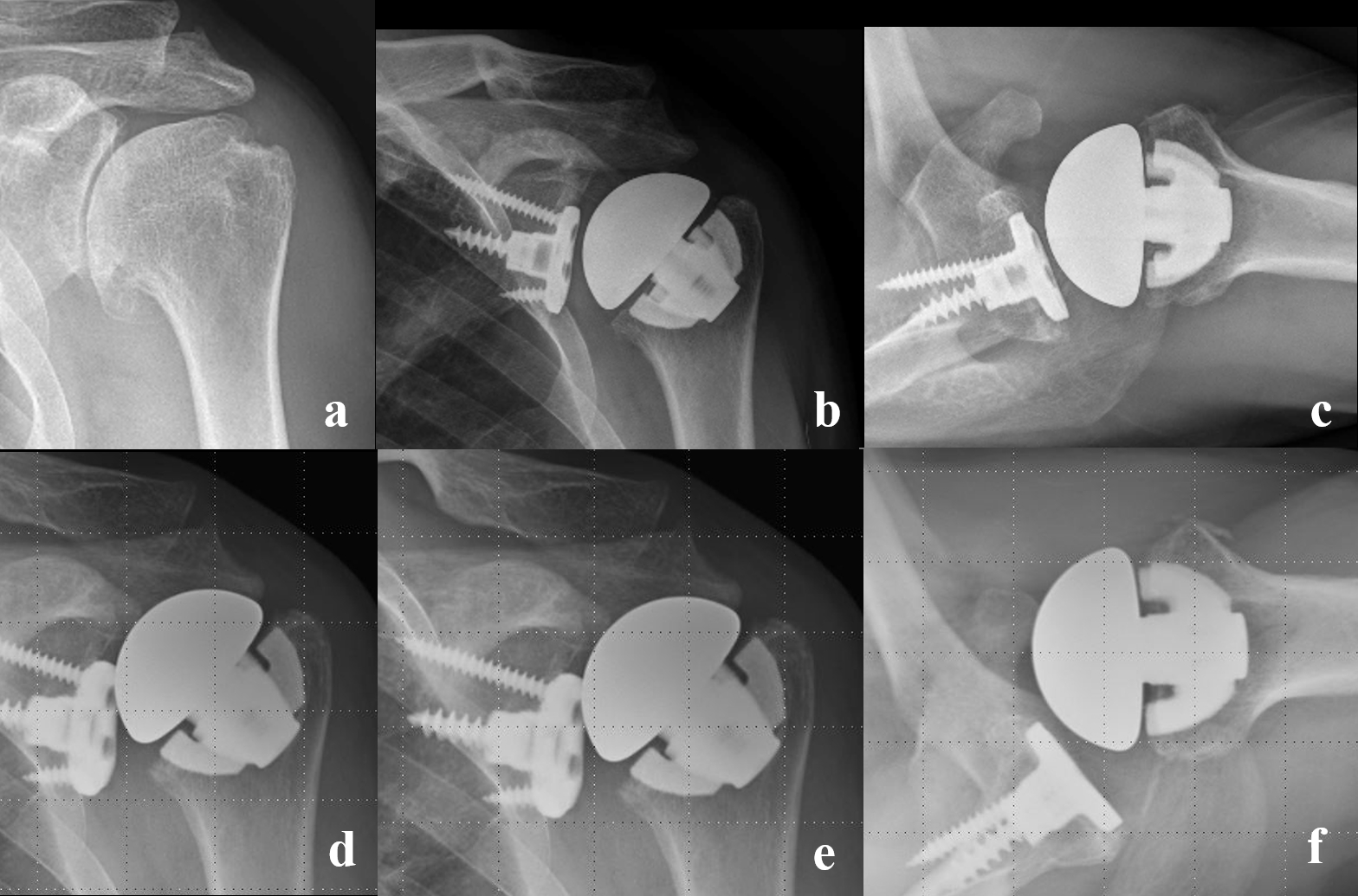
**

(a) Preoperative osteoarthritic changes were found in the X ray. (b, c) Anatomic total shoulder arthroplasty (aTSA) using the convertible metal-backed glenoid with stemless stem was performed. (d, e, f) During follow-up, subscapularis failure was confirmed by radiographic images.

**Figure 2**

**
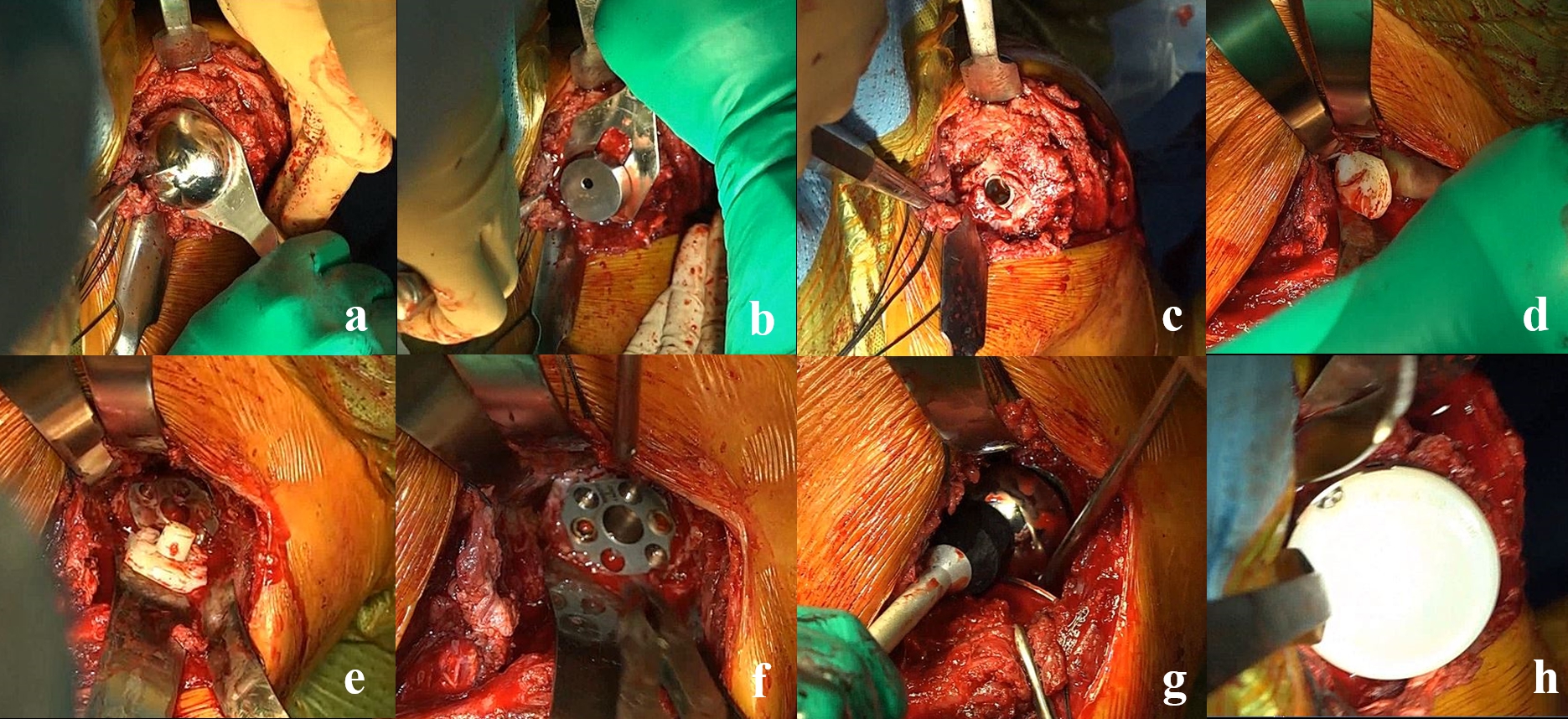
**

(a-c) On conversion to revere total shoulder arthroplasty (rTSA), humeral head was first retracted. (d-f) Then, the polyethylene (PE) inserted on the MB baseplate was removed using an osteotome or chisel while retaining the MB baseplate. (g) Glenosphere was inserted on retained MB baseplate. (h) Then, the humeral tray with PE was changed.

**Figure 3**

(a-c) Postoperative radiographic images after conversion of aTSA to rTSA were shown.


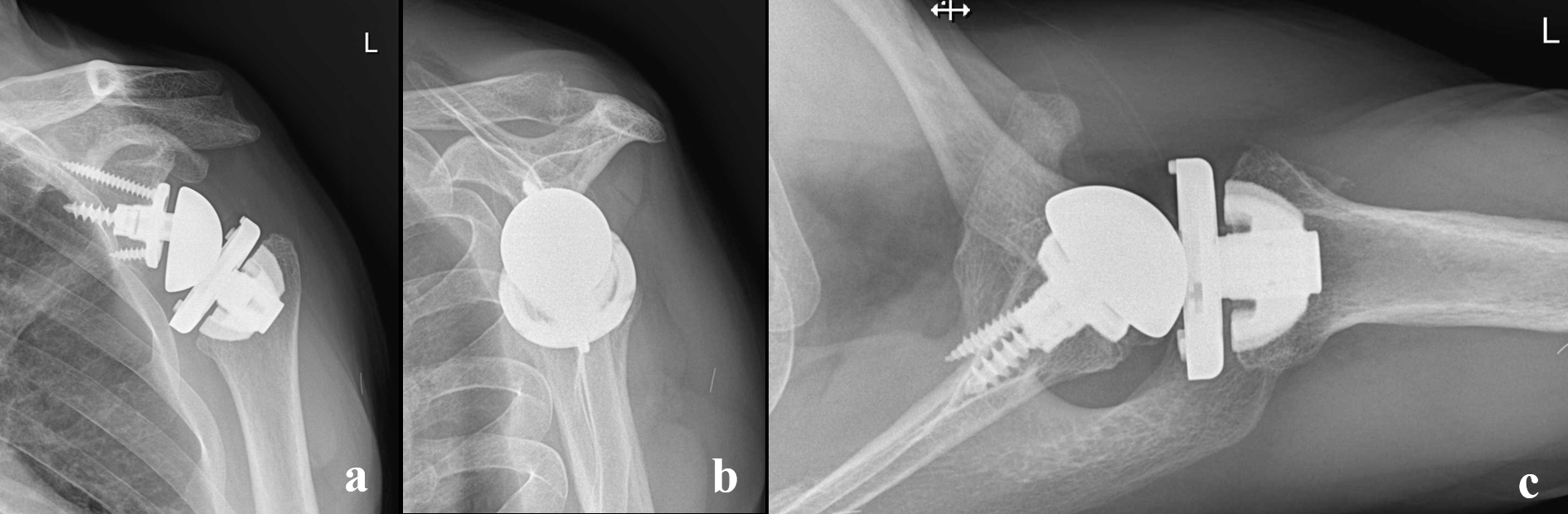

Supplement: Supplementary file 1 — Additional File 1: In the case of converting anatomical total shoulder arthroplasty (aTSA) to reverse total shoulder arthroplasty using a convertible metal-backed (MB) glenoid component system due to aTSA failure, the humeral head and polyethylene (PE) were easily removed without causing any further damage to the glenoid bone. Subsequently, the glenosphere was easily inserted onto the retained MB base plate, and the humeral tray with PE was replaced without any difficulties. [file 12893_2023_2092_MOESM1_ESM.docx]
